# Analysis of Compound Synergy in High-Throughput Cellular Screens by Population-Based Lifetime Modeling

**DOI:** 10.1371/journal.pone.0008919

**Published:** 2010-01-27

**Authors:** Martin Peifer, Jonathan Weiss, Martin L. Sos, Mirjam Koker, Stefanie Heynck, Christian Netzer, Stefanie Fischer, Haridas Rode, Daniel Rauh, Jörg Rahnenführer, Roman K. Thomas

**Affiliations:** 1 Max Planck Institute for Neurological Research with Klaus-Joachim-Zülch Laboratories of the Max Planck Society and the Medical Faculty of the University of Köln, Max Planck Society, Köln, Germany; 2 Department of Statistics, Technical University of Dortmund, Dortmund, Germany; 3 Department I of Internal Medicine and Center of Integrated Oncology Köln – Bonn, University of Köln, Köln, Germany; 4 Chemical Genomics Center of the Max Planck Society, Max Planck Society, Dortmund, Germany; Dr. Margarete Fischer-Bosch Institute of Clinical Pharmacology, Germany

## Abstract

Despite the successful introduction of potent anti-cancer therapeutics, most of these drugs lead to only modest tumor-shrinkage or transient responses, followed by re-growth of tumors. Combining different compounds has resulted in enhanced tumor control and prolonged survival. However, methods querying the efficacy of such combinations have been hampered by limited scalability, analytical resolution, statistical feasibility, or a combination thereof. We have developed a theoretical framework modeling cellular viability as a stochastic lifetime process to determine synergistic compound combinations from high-throughput cellular screens. We apply our method to data derived from chemical perturbations of 65 cancer cell lines with two inhibitors. Our analysis revealed synergy for the combination of both compounds in subsets of cell lines. By contrast, in cell lines in which inhibition of one of both targets was sufficient to induce cell death, no synergy was detected, compatible with the topology of the oncogenically activated signaling network. In summary, we provide a tool for the measurement of synergy strength for combination perturbation experiments that might help define pathway topologies and direct clinical trials.

## Introduction

The vision of personalized cancer medicine has recently become an achievable goal through the development of novel cancer therapeutics and the link of their efficacy to somatic genetic aberrations (or, “lesions”). Prominent examples are *ERBB2*-amplified breast cancers [1] that respond to ERBB2 inhibition, BCR-ABL-translocated chronic myeloid leukemia patients that can be successfully treated with the ABL kinase inhibitor imatinib [2], [3], or *EGFR*-mutant non-small cell lung cancers (NSCLC) that are sensitive to treatment with the EGFR inhibitors erlotinib and gefitinib [4]. However, the enthusiasm about this success has been dampened by limited tumor shrinkage in most patients and the occurrence of relapse after an initial response [5], [6], [7], [8], [9], [10].

The concept of simultaneous targeting of more than one signaling pathway or pathway component has been pursued for many years as a promising strategy to increase treatment efficacy or prevent the emergence of drug resistance [11], [12], [13]. In the area of conventional cytotoxic chemotherapy, only the combination of multiple drugs has enabled actual cures for leukemia and lymphoma patients [14]. Additional examples include the successful combination of therapeutic antibodies and chemotherapy for treatment of lymphomas, as well as breast and colorectal cancer [15], [16]. Finally, combining specific inhibitors of oncogenic signaling pathways has resulted in highly synergistic treatment responses in clinically relevant tumor models [17], [18], [19]. Thus, systematic approaches to interrogate synergistic compound combinations and to link these to individual genetic lesions are required to move these combinations into clinical trials more rapidly. Another notion supporting the systematic study of such combination therapies comes from the careful biochemical dissection of oncogenic signaling pathways: it was shown that most of these pathways are interconnected by feedback loops [20], [21], [22]. Thus, simultaneously blocking two or more of such pathways might lead to activation of the alternate pathway by release of negative feedback loops. Accordingly, beyond the obvious benefit for drug discovery, such studies may help defining signaling pathway topology connected with individual genetic lesions.

Unfortunately, establishing synergistic compound combinations at greater scale is typically hampered by the necessity to screen multiple compound concentrations of one compound against different concentrations of another compound. Furthermore, many analytical approaches do not consider continued proliferation of viable cells and do not afford establishing statistically meaningful representations of screening data across a broad experimental range.

Several methods for the detection of compound synergy have been proposed [23], [24], [25], [26]. In summary, the diverse definitions of synergy and methods for its detection are based on two principles: Loewe additivity [27] and Bliss independence [28]. However, a precise methodological derivation of the analytical procedure and the close adaptation to an experimentally tractable setup amenable to high-throughput cellular screening have been lacking so far. We therefore set out to develop both a novel approach for high-throughput cell-based screening of multiple compound concentrations and a statistical framework to define synergy as a probabilistic lifetime process under single and combined chemical perturbations. We applied this model to screening data derived from a screen of a panel of genetically and phenotypically characterized NSCLC cell lines and determined global genetic settings in which synergy of the irreversible EGFR/ERBB2 inhibitor BIBW-2992 and a dual PI3K/mTOR inhibitor PI-103 is most pronounced.

## Results

### Population-Based Analysis of Cell Viability Measurements

We reasoned that cellular dose response that is commonly used for cell viability measurements is based on a change of the cellular growth rate when a given perturbation (in most cases, a chemical compound) is added in comparison to untreated cells. This description allows a probabilistic interpretation in terms of a stochastic waiting-time process. For a given compound concentration *x*, these ideas lead to the following relationship
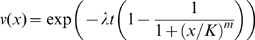
(1)where *v* is the modeled viability, *t* is the time at which the measurement has been carried out, and λ, *K*, *m* are the model parameters. Equivalently, the model can be interpreted such that each cell in the population has an exponentially distributed lifetime after the treatment. As rate of the exponential distribution we then obtain 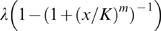. In case of dual-specificity inhibitors (i.e., inhibitors inhibiting more than one target), sensitivity of both targets might be very distinct. It may happen that one target is already completely inhibited with the lowest concentration in the screen. To capture this effect, an offset 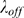, 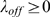 can be added to the model, leading to the rate 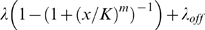. Details of the mathematical model and its derivation are presented in the **Supplementary Note S1**.

**Figure 1:**
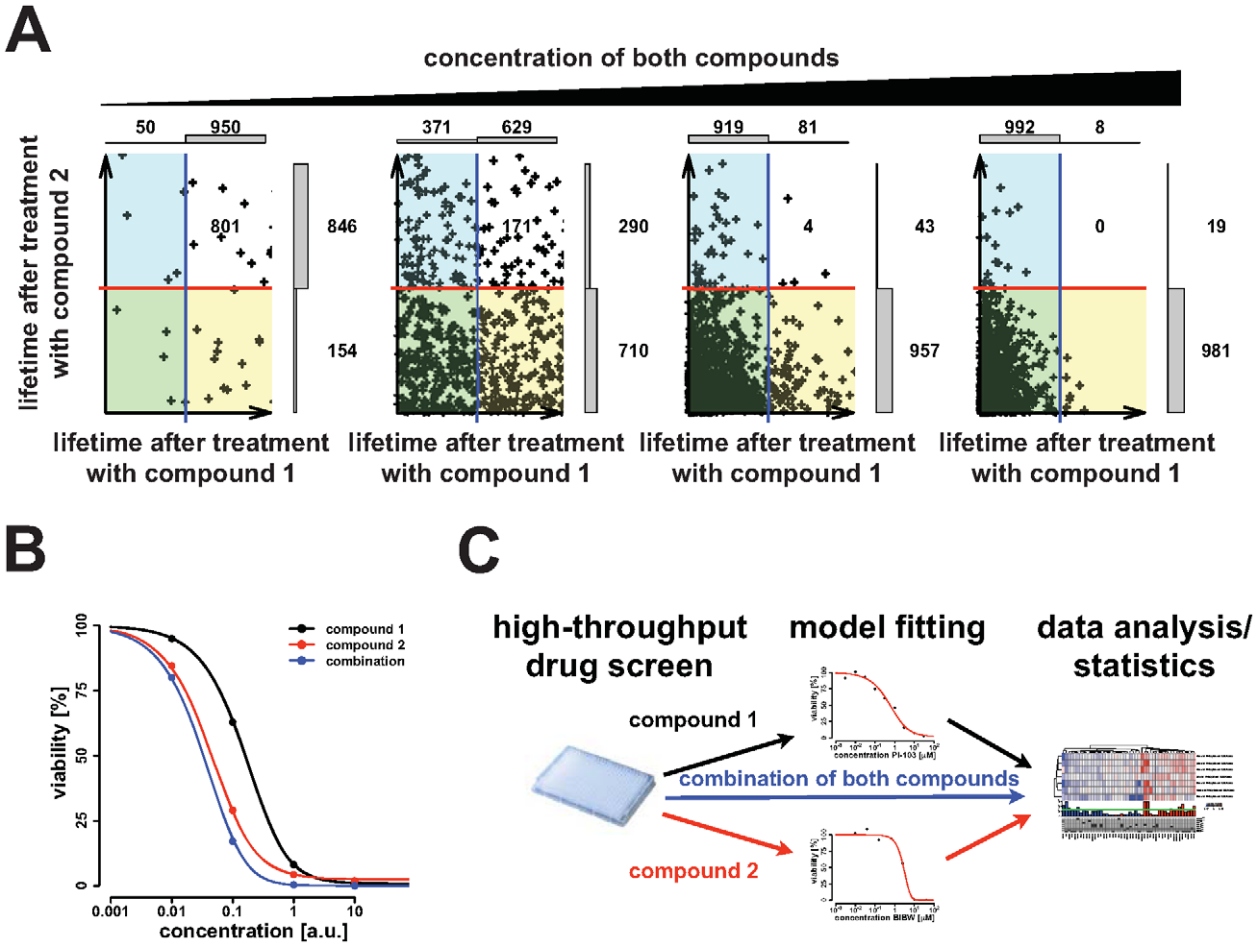
Overview of the model and method to detect synergistic compound combinations. (**A**) Model based simulation of the lifetime of 1000 cells after treatment. The x-axis corresponds to the lifetime after treating cells with compound 1 and the y-axis shows the lifetime after treatment with compound 2. Concentrations of both compounds are increased by a factor of 10 from left to right. Either the vertical blue line in case of compound 1 or the horizontal red line for compound 2 indicates time of measurement. Thus, the number of viable cells at measurement is given by the number of data points on the right side of the blue lines (after treatment with compound 1) or above the red line (in case of compound 2). Distributions of viable and dead cells are displayed by bars at the upper and right side of each panel. Combining both compounds and assuming that the combination of both compounds is neither synergistic nor antagonistic yields a certain number of viable cells that is represented by dots in the white area. This notion reflects the fact that the minimal lifetime between the two compounds (x and y-axis) has to be taken for the combination. (**B**) Theoretical dose response curves are shown for the previous example. Data points were computed from results of the simulation shown in (A). Even for the relatively small population of 1000 cells, the simulated data points and the theoretical curve match. (**C**) Scheme of the procedure to detect synergistic and antagonistic compound combinations. Starting from a high-throughput compound screen, the model is fitted to all single-agent measurements. From the fitted model parameters, curves are computed for each combination separating synergy from antagonism. Measured data of the combination screen are then compared to the computed curves and finally analyzed using a rank-based statistical test.

**Figure 1A** shows the simulated individual lifetime of 1000 cells, which have been treated with two different compounds. Compound concentrations increase from the left to the right panels. Blue and red lines indicate the time of measurement and data points which are located at the yellow and white area represent cells which are still viable at the time of measurement when treated with compound one. Data points falling into the blue and white areas display viable cells after treatment with compound two. In case of a non-synergistic and non-antagonistic compound combinations the lifetime of the cells is given by the smallest lifetime when treated with either compound (white area). Translating the idea of “minimal lifetime” into a mathematical model leads to a product of the two single compound dose response curves modeled by **Eq. (1)** as non-synergistic combined effect **(** **Fig. 1b** **, blue curve)**; this concept is compatible with Bliss independence. A simulation over a relatively small population on 1000 cells revealed that the simulated points closely correspond to the theoretical curves **(** **Fig. 1b** **)**.

With this mathematical model we next sought to distinguish between synergy and antagonism of compound response curves derived from high-throughput screening efforts **(** **Fig. 1C** **)**. Starting from the high-throughput screening platform dose response curves from both single compounds as well as their combinations were determined for a large panel of genetically annotated non-small cell lung cancer cell lines. **Equation (1** **)** is then fitted to the dose response curve of each single compound screen. This yields the model parameters λ, *K*, *m*, from which the curve separating synergistic from antagonistic compound combinations can be computed according to **Eq. (S10)** of the **Supplementary Note S1**. For a given compound combination, the difference between the computed curve and the measurement is then a measure for synergy or antagonism, respectively. This measure is denoted by synergy strength. Due to the presence of noise, several different compound combinations are needed to filter out cell-lines, which show significant enrichment of synergy strength over different combinations. To this end, a rank sum approach is used. In order to account for multiple hypothesis testing the false-discovery rate (FDR) framework [29] was applied.

### Applying the Model for Single Compound Screen of PI-103 and BIBW-2992

**Figure 2:**
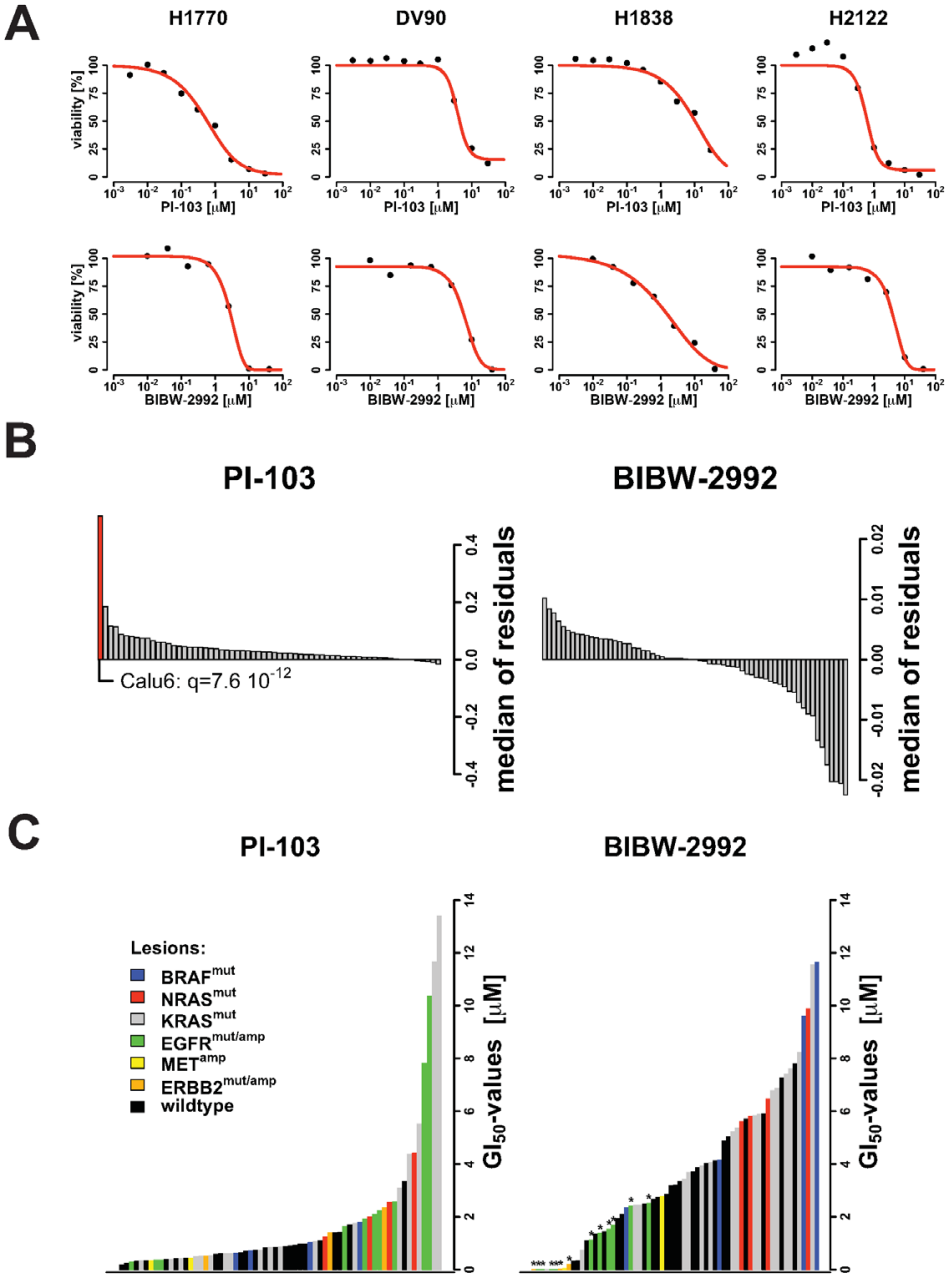
Results obtained from the single-compound screen of PI-103 and BIBW-2992. (**A**) Kill-curves are exemplarily shown for two compounds (PI-103 and BIBW-2992) and 4 cell lines. Solid red lines display the fitted model to the measured data shown by black points. (**B**) Analysis of the model residuals (i.e., difference between the measurements and the model prediction) for both compounds and each cell line. Shown are the distributions of the residuals' medians over the screened concentrations. A statistical test to detect significant outliers reveals that only the cell line Calu3 when screened against PI-103 is not compatible with the distribution of the median of residuals (FDR q-value = 7.6 10^−12^); highlighted by a red bar. (**C**) Profiles of GI_50_-values for PI-103 and BIBW-2992. GI_50_-values were computed using the proposed model and sorted according to the sensitivity of the cell line to the inhibitions: most sensitive cell lines are on the left side and most resistant cell lines are shown on the right side. Colors symbolize most common genomic alterations in NSCLC. In case of EGFR^mut/amp^ and ERBB2^mut/amp^ a genomic alteration can either be a mutation or a gene copy number amplification (≥4 copies are considered as alteration), for MET^amp^ only amplifications are reported, the remaining alterations, BRAF^mut^, NRAS^mut^, KRAS^mut^ are mutations. For BIBW-2992, asterisks highlight those cell lines, which harbor lesions either in EGFR or ERBB2.

In order to validate the proposed model, **Eq. (1)**, we screened 65 of the 84 non-small cell lung cancer cell lines [9] against the irreversible EGFR/ERBB2 inhibitor BIBW2992 and the PI3K/mTOR inhibitor PI-103. We selected 4 out of the 65 cell lines and fitted the dose response curves to the corresponding data points **(** **Fig. 2A** **)**. We next determined the difference between the viability predicted by the model and the experimentally determined values (model residuals). To assess the quality of the model we computed the median of the residuals over the concentrations for each compound and cell line **(** **Fig. 2B** **)**. For both compounds, significant outliers are then identified under the assumption that the medians of the residuals are normally distributed around zero. Using a 5% level of significance and after correcting for multiple testing we identified only one outlier: Calu6 screened with PI-103 (FDR q-value = 7.6 10^−12^). However, this outlier can safely be neglected since it did not distort the following analysis. In summary, the proposed model fits well to the measured data and is therefore a suitable basis for the identification of synergistic compound combinations.

Computing half-maximal-inhibitory concentrations **(Eq. (2)** **,** **Materials and Methods** **)** for PI-103 and BIBW-2992 **(** **Figure 2C** **)** shows no clear association between the genomic lesions and the single-agent activity of PI-103 with the used cell proliferation assay [19]. As expected, in the case of the irreversible EGFR/ERBB2 inhibitor BIBW-2992, cell lines dependent on EGFR and ERBB2 signaling (due to the presence of drug-sensitizing genetic alterations in these genes) are substantially enriched in the highly sensitive cell lines [30], [31].

### Application of the Model for Combinational Compound Screen of PI-103 with BIBW-2992

**Figure 3:**
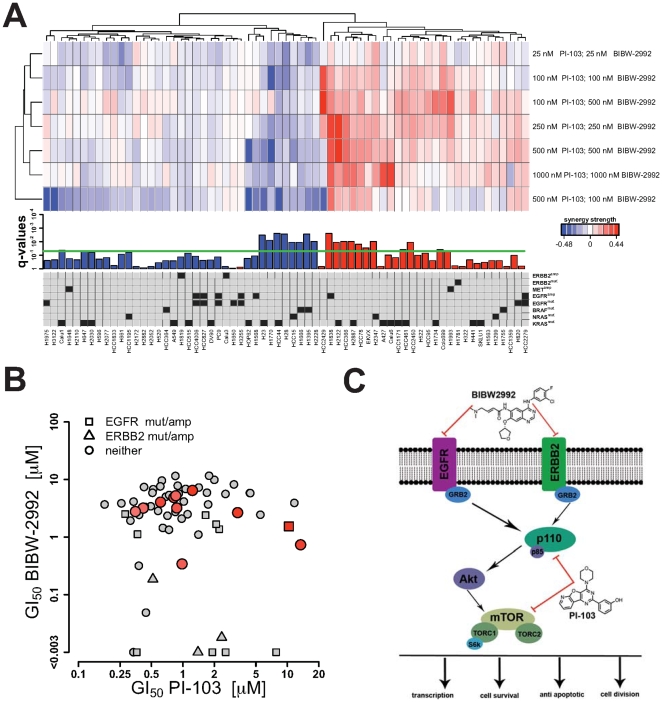
Exploring synergy and antagonism of the compound combination PI-103 with BIBW-2992. (**A**) Combinations of PI-103 and BIBW-2992 were screened for all cell lines and the synergy strength score was computed as difference between measured data and the curve separating synergy from antagonism. Hierarchical clustering clearly classifies the cell lines into two groups according to the algebraic sign of the synergy strength score (positive: synergy; negative: antagonism). Results of a rank sum-based statistical test mainly reproduce the results from cluster analysis. Setting the level of significance to a false discovery rate of 5% (horizontal green line) yields 11 cell lines showing synergy. Finally, the annotation of 8 frequent genomic aberrations indicates that almost all cell lines harboring genomic alterations in ERBB2 family member do not benefit from the combination. (**B**) Shown is the relationship between single-agent GI_50_-values and synergy. All cell lines showing significant synergy are highlighted by red symbols. Annotating the cell lines with mutation and copy number status of EGFR and ERBB2 (distinguished by quadratic symbols and triangles) confirms the previous finding that cell lines harboring alterations in EGFR/ERBB2 do not significantly benefit from the combination in terms of synergy. (**C**) Shown are the main signaling network compounds downstream EGFR and ERBB2 as well as the targets of BIBW-2992 and PI-103. Since the PI3K-mTOR pathway is downstream EGFR/ERBB2, cell lines which depend on EGFR/ERBB2-signaling do not benefit from the combination.

In order to test the accuracy of our model to detect synergy of compound combinations we next sought to systematically assess the viability of cells treated with a combination of the two compounds. With the EGFR/PI3K signaling cascade being one of the most frequently mutated pathways in lung cancer, we speculated that combined inhibition of EGFR- and PI3K/mTOR-signaling might be effective in our cell line panel of NSCLC cells. The presence of considerable experimental noise **(** **Fig. 2B** **)** makes it necessary to test different combinations for the determination of synergy. Therefore, seven compound dose combinations of PI-103 and BIBW-2992 were applied for the 65 cell lines already used in the single screens. The curve, which separates synergistic from antagonistic combinations, is computed from the previously determined fits, which serves as basis for the synergy strength. We next computed this synergy score and applied hierarchical clustering to the data matrix of the synergy strength **(** **Fig. 3A** **)**. This analysis revealed two distinct groups, separating cell lines according to synergistic and antagonistic behavior. To assess which cell lines in those clusters display a significantly synergistic or antagonistic response to combined EGFR-PI3K inhibition, we employed a rank sum-based statistical test **(** **Fig. 3A** **)**. Ranks of synergy strength were computed over all cell lines but for each measured combination separately and summed over the seven combinations. Next, a statistical test was derived to test if high or low ranks were enriched. To correct for multiple testing all p-values were corrected using the false-discovery rate approach. Resulting q-values are shown in **Fig. 3A**, where the horizontal green line indicates the chosen 5% false-discovery rate cutoff. We identified 11 cell lines, for which combined PI-103/BIBW-2992 treatment was significantly synergistic. Our analysis revealed that cell lines harboring either amplification or a mutation in either EGFR or ERBB2 were not enriched in the fraction of cell lines responding in synergistic fashion to the combination of both compounds. These results suggest that inhibition of ERBB-signaling in these cell lines is already sufficient to effectively shut down survival signaling. However, no other significant correlation between synergy strength and genotype could be observed **(** **Fig. 3A** **)**.

To further validate our methodological framework, we compared our results with synergy predictions based on the combination index method [12], [32], [33]. While the combination index yielded a result in only 66% of the screening data analyzed, our approach yielded robust synergy scores across the entire data set, thereby affording application to high-throughput screens. However, in the fraction of data that could be analyzed by both methods, synergistic cell lines determined with our method and the combination index method largely overlapped **(Fig. S1)**. This is underscored by a regression analysis between the negative-log-transformed combination index and the synergy strength score **(Fig. S2)**, which showed a significant positive correlation (r^2^ = 0.45; p<10^−6^). The enhanced robustness of our approach is largely due to the fact that it takes into account the entire dose response relationship and is not restricted to the behavior of a single point **(Supplementary Note S1)**.

The observation that combination treatment is not beneficial in cell lines with oncogenic alterations in EGFR and ERBB2 indicates that there might be a relationship between activity of the individual compounds and synergy. In order to demonstrate such a relationship, we plotted the GI_50_-values of PI-103 against those of BIBW-2992 and labeled all data points of cell lines with genetic aberrations in the EGFR/ERBB2 receptor tyrosine kinases **(** **Fig. 3B** **)**. This analysis recapitulated the previous findings that cell lines, which are primarily dependent on EGFR/ERBB2 signaling (GI_50_<0.1 µM), do not benefit from the combination of ERBB/PI3K-pathway inhibition. Remarkably, our findings are in line with the general topology of the signaling pathways downstream of EGFR and ERBB2 **(** **Fig. 3C** **)**. Since oncogenically activated EGFR and ERBB2 receptors preferentially signal through the PI3K pathway [19] combined blockade of those pathways is not expected to be synergistic for cells depending on EGFR or ERBB2. In other words, potent inhibition of strong oncogenic signals *upstream* is already sufficient to induce apoptosis, independent of the inhibition of further components *downstream* **(** **Fig. 3C** **)**. The same seems to be valid for three cell lines with the lowest PI-103 GI_50_-values. However, dependency on PI3K-mTOR signaling was generally less pronounced (expressed by higher GI_50_-values) which might be a result of alternative pathways upstream PI3K such as the mitogen-activated protein kinase (MAPK) and feedback loops connecting the two pathways [19]. However, synergistic combinations clustered around a GI_50_-value of 1µM for PI-103. We therefore speculate that a supra-threshold activity of PI3K inhibition is needed to obtain synergy.

In order to provide a deeper characterization of the genotypes, we extended the previously used genetic annotation with significant copy number aberrations computed by GISTIC [34]. A complete list of all identified copy number aberrations and the mutation status of 7 genes is given for the cell lines showing synergistic behavior in **Table S1**. Similar to the analysis done in [9], we performed a k-nearest-neighbor prediction on this data set and found no significant predictor of synergy **(Table S2)**. The inability to predict synergy from genetic lesions is probably hampered by the necessity to restrict the analysis to recurring and highly focal copy number lesions as identified by GISTIC and the focus on the most frequent gene mutations in NSCLC.

## Discussion

Starting from general considerations about cell viability measurements, we derived a model for inferring cell survival curves from high-throughput cell-based screening data [35]. This model laid the basis for detection of synergy strength of compound combinations. Here, the central assumption is that the median-effect equation [12], [32], [33] is coupled linearly to a cell-killing rate under treatment. Validation of the model in a panel of 65 lung cancer cell lines perturbed using PI3K and EGFR/ERBB2 signaling pathway inhibitors revealed general rules of the signaling pathway topology downstream of genetically altered EGFR and ERBB2 kinases. Thus, our approach affords analysis of synergy of compound combinations in high-throughput cell-based screens in scalable fashion.

Other approaches involving the network structure of complex biological systems have been proposed [24], [35]. Our model has the advantage of permitting systematic statistical analyses of synergy employing generic laboratory cellular screening experiments involving a vast array of genetic cellular backgrounds. Another major advantage of our model is its stochastic nature describing the lifetime of cells under treatment. This allows a rigorous derivation of a synergy score when cells are treated with a combination of compounds. In fact, we confirmed Bliss independence [28] based on this computation but within a solid theoretical framework.

As application of the proposed analytical framework, we applied the method to single and combined screens of the PI3K inhibitor, PI-103 and the EGFR/ERBB2 inhibitor, BIBW-2992. Our model captured previous findings that genetic alterations in EGFR are predicting sensitivity of EGFR inhibitors [4], [36], [37]. Analysis of synergy between PI-103 and BIBW-2992 revealed that cell lines dependent on EGFR/ERBB2-signaling do not benefit from the combination **(** **Fig. 3A, B** **)**, which is in line with the network topology suggesting a preferential linear downstream engagement of PI3K signaling downstream of oncogenically activated receptor tyrosine kinases [7], [19]. Previous work carried out in transgenic EGFR and ERBB2-mutant mice showed substantial tumor regression when mice were treated with a combination of BIBW-2992 and rapamycin targeting mTOR (or more specifically TORC1) [30], [31]. However, both transgenic alleles in these studies impair binding of quinazoline-based EGFR inhibitors, thus resulting in inefficient target inhibition [38]. Thus, adding downstream inhibition in the setting of incomplete upstream target inhibition can result in synergy, even though the pathway itself is linear **(** **Fig. 3C** **)**. Here, crosstalk or an upstream branching into other signaling components can mediate such an effect. In our study, signaling through the MAPK pathway might substantially contribute to synergy since there are numerous interconnections between MAPK and PI3K signaling pathways.

In summary, we introduced a new methodological framework to detect synergy of compound combinations across a large panel of cancer cell lines. The analysis of a first combination screen supported a view of a mostly linear signaling pathway topology downstream of oncogenically activated EGFR/ERBB2 kinases [19]. Thus, beyond enabling high-throughput analyses of compound combinations, our approach affords general insights into pathway functionality and pathway interrelations.

## Materials and Methods

### Cells

The used cell line collection was previously described in [9]. Cells were routinely controlled for infection with mycoplasm by MycoAlert (www.cambrex.com) and were treated with antibiotics according to a previously published protocol [39] in case of infection.

### Cell-Based Screening

All compounds were purchased from commercial suppliers or synthesized in house, dissolved in DMSO and stored at −80°C. Cells were plated into sterile microtiter plates using a Multidrop instrument (http://www.thermo.com) and cultured overnight. Compounds were then added in serial dilutions. Cellular viability was determined after 96h by measuring cellular ATP content using the CellTiter-Glo assay (www.promega.com). Plates were measured on a Mithras LB940 plate reader (www.bertholdtech.com).

### Copy Number Analysis

Genomic DNA was extracted from cell lines using the PureGene kit (www.gentra.com) and hybridized to high-density oligonucleotide arrays (Affymetrix, Santa Clara, CA) interrogating 238,000 SNP loci on all chromosomes except Y, with a median intermarker distance of 5.2 kb (mean 12.2 kb; http://www.affymetrix.com). Array experiments were performed according to manufacturer's instructions. SNPs were genotyped by the Affymetrix Genotyping Tools Version 2.0 software. We applied GISTIC [34] to analyze the data set. The GISTIC algorithm was run using a copy number threshold of 4 in case of amplifications and 1 for deletons. To ensure compatibility of the copy number data with mutation data we dichotomized copy numbers with the following thresholds: 4 for amplifications and 1 for deletions.

### Model Based Computation of GI_50_-Values

Applying the half-maximal-inhibitory concentration concept (“GI_50_-values”) we set the viability to 50% in Eq. (1); followed by a few algebraic rearrangements yields the model-based computation of the GI_50_-values:
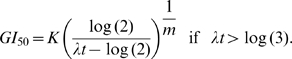
(2)


Positivity of the GI_50_-values is guaranteed by the condition in Eq. (2). If this condition is not satisfied, no GI_50_-value exists, i.e., the on-target inhibition is to weak to kill enough cells such that a viability of 50% can be reached.

### Data Analysis and Statistics

The model of single-agent kill curves, Eq. (1), are fitted to data. To this end, a maximum likelihood approach is employed to estimate the model parameters λ, *K*, *m*. This requires non-linear optimization; we chose the Levenberg-Marquardt method for this optimization [40], [41]. P-values where corrected for multiple testing using the false-discovery-rate approach [29]. The p-value adjustment as well as the cluster analysis was carried out in R version 2.7.1 (http://www.R-project.org).

### Rank Sum Rest

We decided to employ a rank sum based approach to provide a statistical measure for synergy. This approach has the advantage that it also takes prevalence across different cell lines into account and does not purely rely on the synergy strength. This is an important and therapeutically relevant property of the statistical test.

Let us consider the synergy strength measure: 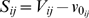, where 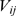 is the measured viability for the combination 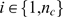 and cell line 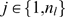. The computed curve separating synergy from antagonism, given by the product of both single compound dose response curves (**Eq. (S10), Supplementary Note S1**), is denoted by 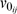. Ranks are computed over all cell lines *j* but for each combination *i* separately; resulting in the rank matrix 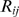. Utilizing that the ranks are uniformly distributed leads to the following variance of the ranks across the cell lines:
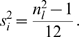



Moreover, under the null-hypothesis that there is no association between the ranks of each combination, the variance of the rank sum 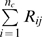 is
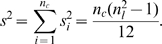
(3)


Relating the rank sum to the median is not useful in our case. If, e.g., 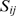 is negative for all *i* and *j* (i.e., there is no sample showing synergistic behavior), a median centered rank sum test would assign a few samples as being synergistic. To derive the test statistics, which corrects for such an effect, we relate the rank sum to the rank where the synergy score 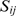 changes its sign. To this end, we compute for each concentration *i* the rank that has the lowest absolute synergy score
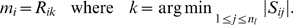



Finally, the rank sum statistics we propose to test for synergy is given by
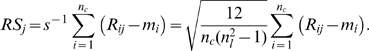
(4)


Under the null-hypothesis that there is no association between the ranks of different concentrations and that the synergy score fluctuates around zero, the distribution of 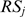 can be approximated by a standard normal distribution. This approximation is asymptotically (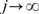) correct and used in our analysis.

## Supporting Information

Supplementary Note S1 Analysis of compound synergy in high-throughput cellular screens by population-based lifetime modeling.
(0.08 MB PDF)

Figure S1 Comparison between the combination index method and the method we propose. Shown is the clustered matrix of the synergy strength measure, as in Fig. 3A, together with the combination index. Significantly synergistic cell lines which where detected with our method are highlighted by red bars. Missing bars indicate that for those cell lines the computation of the combination index was not possible.
(0.69 MB PDF)

Figure S2 Correlation analysis between both methods. To adapt the scale of both measures, we performed a transformation of the combination index using the negative logarithm. The regression line is displayed by the straight red line. Moreover, we found a significant positive correlation (r^2^ = 0.45; p<10^−6^), which confirms that both methods follow the same trend.
(0.12 MB PDF)

Table S1 Genomic annotation of all 11 cell lines showing synergistic behavior. Significant copy number regions were identified using GISTIC. To assure comparability with mutation data, copy numbers were dichotomized with the following thresholds: 4 in case of amplifications and 1 for deletions.
(0.03 MB PDF)

Table S2 Multi-lesion predictor of synergy tested with the KNN method, Fishers exact test and t-test are displayed; here, only p-values smaller than 5% are shown. The Youden-Index (i.e., sensitivity+specificity-1) of zero indicates that the result has no predictive power.
(0.02 MB PDF)
