# Supplementary material for: Analysis of Compound Synergy in High-Throughput Cellular Screens by Population-Based Lifetime Modeling
Source: PLoS One. 2010 Jan 27;5(1):e8919. doi: 10.1371/journal.pone.0008919 (PMC2811738; doi:10.1371/journal.pone.0008919)
Supplement: Supplementary Note S1 — Analysis of compound synergy in high-throughput cellular screens by population-based lifetime modeling. (0.08 MB PDF) [file pone.0008919.s001.pdf]

Supplementary Note
Analysis of compound synergy in high-throughput cellular screens by population-based lifetime modeling

Martin Peifer, Jonathan Weiss, Martin L. Sos, Mirjam Koker, Stefanie Heynck, Christian Netzer, Stefanie Fischer, Haridas Rode, Daniel Rauh, Jörg Rahnenführer and Roman K. Thomas

Mathematical model

Model for the single-agent dose response curve

To derive a suitable model for the dose response curve under treatment of a single compound, we assume that the cells are in the exponential growth regime. For the used assay, this is justified because over the period of 96 hours the growth of the cells is neither limited by nutrition nor by space. Without addition of compounds, the number of viable cells at time t is given by the exponential relationship $N(t) = N_0 \exp(\alpha t)$, where N_0 is the total number of cells just after the experiment has started. If compound is added, the growth rate is changed to $\tilde{\alpha}$, where $\tilde{\alpha} < \alpha$. Therefore, the number of viable cells under treatment is given by $\tilde{N}(t) = N_0 \exp(\tilde{\alpha} t)$. Since the measured luminescence signal produced by the CellTiter-Glo Assay is proportional to the number of viable cells, the intensity $I(t) = I_0 N(t)$ is measured for the untreated cells and $\tilde{I}(t) = I_0 \tilde{N}(t)$ for the cells for which the compound has been added. Viability v is then given by the quotient of $I(t)$ by $\tilde{I}(t)$, which yields

$$v = \frac{\tilde{I}(t)}{I(t)} = \exp\left(-(\alpha - \tilde{\alpha})t\right) \ . \tag{S1}$$

As next step, the effect of the compound is linked to the rate $\tilde{\alpha}$. Using the median-effect equation [2, 4, 3], the inhibition strength $S(x)$ at compound concentration x is given by

$$S(x) = \frac{1}{1 + (x/K)^m} \ , \tag{S2}$$

where K can be interpreted as binding affinity and m is the Hill-coefficient. The strength of the median-effect equation is that its derivation is based on meaningful biochemical

principles, such as the mass-action law. Finally, we assume that rate $\tilde{\alpha}$ is linearly related to the strength of the inhibition $S(x)$, thus $\tilde{\alpha} = \beta S(x) + \gamma$. Inserting this dose-growth rate relationship into eq. (S1), we obtain

$$v(x) = \exp\left(-(\alpha - \beta S(x) - \gamma)t\right) . \tag{S3}$$

At compound concentration zero the viability is always one leading to the condition: $0 = \alpha - \beta S(x = 0) - \gamma$. Because $S(x = 0) = 1$, the parameter set reduces to $\alpha - \gamma = \beta$. If we set $\lambda = \alpha - \gamma$, we obtain the final dose to viability relationship

$$v(x) = \exp\left(-\lambda t\left(1 - \frac{1}{1 + (x/K)^m}\right)\right) . \tag{S4}$$

Probabilistic interpretation

In this paragraph, it is shown that the mathematical model derived above allows a probabilistic interpretation which we borrow from survival analysis. Each cell in the population either shows a response to the compound over the observed period of time t or not. Let T be a random variable at which time a cell shows a response; in our case either cell cycle arrest or apoptosis. Then, the viability v is given by probability that no such event has happened until time point t,

$$v = P(T \geq t) . \tag{S5}$$

For any time point τ with $0 \leq \tau \leq t$, we can introduce a rate of probability λ_0 that a response has occurred. This is given by the following limit

$$\lambda_0 = \lim_{\Delta t \downarrow 0} \frac{P(\tau \leq T < \tau + \Delta t \;| \tau \leq T)}{\Delta t} . \tag{S6}$$

Again, if we assume that there is no limitation in nutrition of the cells and space over the observed time period this rate λ_0 must be constant in time. In survival analysis, the rate of probability λ_0 is known as hazard rate. Using similar computations as, e.g, done in [5], the viability v can be derived from λ_0, resulting in

$$v \;=\; \exp\left(-\int_0^t \lambda_0 \; du\right) = \exp(-\lambda_0 t) . \tag{S7}$$

Note that if the cells are not in an perfect exponential growth regime, the rate λ_0 is time dependent. In this situation, the rate in eq. (S7) has to be exchanged by the time-average $\frac{1}{t}\int_0^t \lambda_0(u) \; du$ and the model still remains valid. Again, coupling the dose effect strength linearly to the rate of probability leads to $\lambda_0 = \lambda\,(1 - S(x))$. Inserting this relationship into eq. (S7), yields the same relation between compound concentration and viability as

previously derived, eq. (S4). The rate of probability that a response occurs is finally given by

$$\lim_{\Delta t \downarrow 0} \frac{P(\tau \leq T < \tau + \Delta t \; | \tau \leq T)}{\Delta t} = \lambda \left(1 - \frac{1}{1 + (x/K)^m}\right) . \tag{S8}$$

If the cells are untreated no compound associated response can happen, this is reflected by fact that the rate of probability vanishes at $x = 0$.

Detecting synergy

Detection of synergy critically depends on the "null-model" separating synergistic compound combinations from antagonistic. For sake of clarity, we only consider combinations of two compounds in the following. A generalization to combinations of more than just two chemical agents can be done in a straight forward manner. In the derivation of the curve separating synergistic from antagonistic combinations (null-model), the probabilistic interpretation given by eqs. (S7) and (S8) plays a crucial role. Let λ_{0_1} be the rate of probability that a response occurs as a result from compound one and λ_{0_2} rate that the response is due to compound two. Correspondingly, T_1 is the time at which compound one responds and T_2 that of compound two. We further define the sets $A = \{\tau \leq T_1 < \tau + \Delta t\}$, $B = \{\tau \leq T_2 < \tau + \Delta t\}$, and $C = \{\tau \leq T_1\} \cap \{\tau \leq T_2\}$, where again $0 \leq \tau \leq t$. The probability that a response at a time point between τ and $\tau + \Delta t$ is either due to compound one or compound two is then $P(A \cup B|C) = P(A|C) + P(B|C) - P(A \cap B|C)$. Since $P(A \cap B|C) = P(A|C) \cdot P(B|C)$ is of second order in Δt its contribution to the rate of probability vanishes in the limit $\Delta t \to 0$. Putting everything together, we obtain the rate of probability for the null-model

$$\lim_{\Delta t \downarrow 0} \frac{P(A \cup B|C)}{\Delta t} = \lim_{\Delta t \downarrow 0} \frac{P(A|C) + P(B|C)}{\Delta t} = \lambda_{0_1} + \lambda_{0_2} . \tag{S9}$$

Let x_1 represent the concentration of compound one and x_2 of compound two. Moreover, the model parameters λ_1, K_1, m_1 are determined from the dose response curve of compound one, and λ_2, K_2, m_2 from the dose response curve of compound two respectively. Applying eq. (S9) to eq. (S7) yields the curve separating synergistic combinations from antagonistic:

$$v_0(x_1, x_2) = \exp\left(-\lambda_1 t \left(1 - \frac{1}{1 + (x_1/K_1)^{m_1}}\right) - \lambda_2 t \left(1 - \frac{1}{1 + (x_2/K_2)^{m_2}}\right)\right) . \tag{S10}$$

If $v(x_1, x_2)$ represents the measured viability of the compound combination, combinations which satisfy $v(x_1, x_2) < v_0(x_1, x_2)$ are synergistic, whereas combinations satisfying $v(x_1, x_2) \geq v_0(x_1, x_2)$ are antagonistic. Since v_0 is the product of the single-agent kill curves, this result is comparable with Bliss independence [1].

Comparison with the combination index method

A frequently used method to assess synergy from drug screening experiments is the combination index method introduced by Chou and colleagues [2, 4, 3]. This method is making use of Loewe additivity [6] to compute an index based on the GI_{50}-values of the single-agent and the combination screen. Note that the GI_{50}-values for the combination screen are lying on a curve (50%-isoline of the dose response surface). To compute the combination index a suitable projection of the dose response surface is usually considered; in the most cases the concentration of one compound is kept fixed while the other is varied. A version of the dose effect equation is fitted to this projection, to estimate the pair of concentrations, c_1 for compound one and c_2 for compound two, where the viability reaches 50% in the combination screen. From the single-compound GI_{50}-values: GI_{50_1} for compound one and and GI_{50_2} for compound two, the combination index CI of mutually independent compounds is then given by

$$CI = \frac{c_1}{GI_{50_1}} + \frac{c_2}{GI_{50_2}} \ .$$

Synergy is predicted when $CI < 1$, antagonism if $CI > 1$, and additivity of both compounds if $CI = 1$.

In order to compare our method with the combination index method, we are faced with the problem that our sampling of the dose response surface is not conform with the projection constraint. Therefore, only five of seven data points from the combination screen could be used determine the combination index. Another restriction comes from the fact, that in some cases the concentrations c_1 and c_2 are outside of the measured range. We decided that in those cases the combination index cannot be computed to prevent model specific extrapolation errors. This restriction forced us to compute the combination index for only 66% of the cell lines.

Figure S1 shows the clustered synergy strength matrix together with results from the combination index analysis. This comparison reveals that both methods share a large overlap and do not lead to contradictory results. However, the computation of the combination index was not possible for most of the cell lines showing antagonistic or additive behavior in our test. Especially the observation that cell lines harboring oncogenic aberrations in EGFR/ERBB2 do not synergistically profit from the combination was not possible using the combination index method in our experimental setting.

To quantify the comparison of both methods, we carried out a linear regression analysis between the rank sum statistics, eq. (4), and the combination index **(Fig. S2)**. In order to adapt the scale of the combination index to that of the rank sum statistics, we transformed the combination index to: $-\log_2(CI)$. The regression analysis confirms that the combination index method shows the same trend than our method. Moreover, we found a significant positive correlation between the methods ($r^2 = 0.45$; $p < 10^{-6}$).

In summary, both methods are sharing the same trend. However, advantages of our method are that it is more flexible in the experimental design, it can still be applied when

the GI_{50}-values lie out of the screened region, and it explores the entire dose response relationship, rather than just at a point.

References

[1] C. I. Bliss. The calculation of microbal assays. *Bacteriol Rev*, 20:243–258, 1956.

[2] T. C. Chou. Derivation and properties of Michaelis-Menten type and Hill type equations for reference ligands. *J Theor Biol*, 59(2):253–276, 1976.

[3] T. C. Chou. Theoretical basis, experimental design, and computerized simulation of synergism and antagonism in drug combination studies. *Pharmacol Rev*, 58(3):621–681, 2006.

[4] T. C. Chou and P. Talalay. Quantitative analysis of dose-effect relationships: the combined effects of multiple drugs or enzyme inhibitors. *Adv Enzyme Regul*, 22:27–55, 1984.

[5] D.R. Cox. Regression Models and Life-Tables. *J R Stat Soc B*, 34(2):187–220, 1972.

[6] S. Loewe. The problem of synergism and antagonism of combined drugs. *Arzneim Forsch*, 3:285–290, 1953.
