# Supplementary figures and images for: Analysis of Compound Synergy in High-Throughput Cellular Screens by Population-Based Lifetime Modeling
Source: PLoS One. 2010 Jan 27;5(1):e8919. doi: 10.1371/journal.pone.0008919 (PMC2811738; doi:10.1371/journal.pone.0008919)

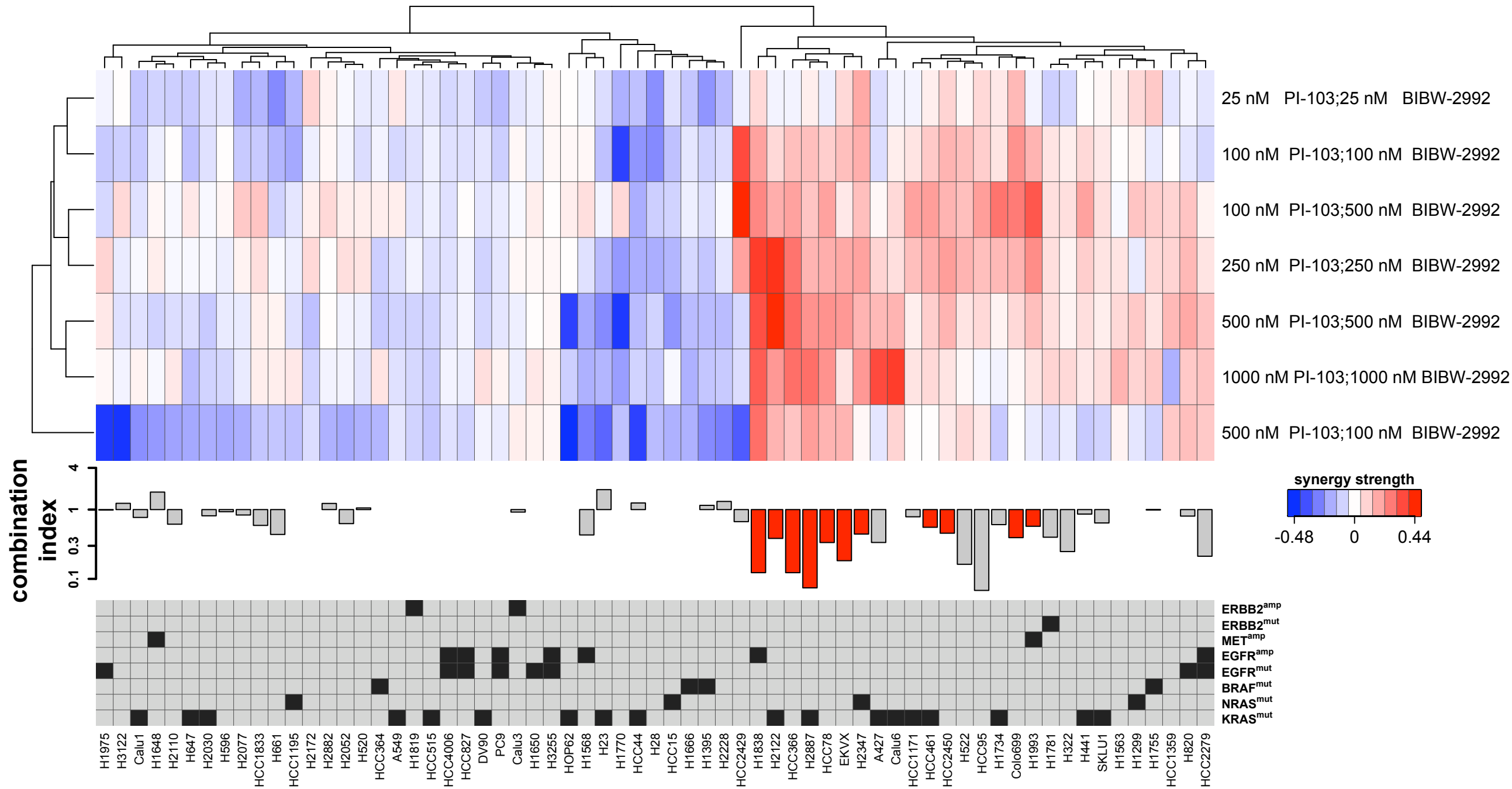

Supplement: Figure S1 — Comparison between the combination index method and the method we propose. Shown is the clustered matrix of the synergy strength measure, as in Fig. 3A, together with the combination index. Significantly synergistic cell lines which where detected with our method are highlighted by red bars. Missing bars indicate that for those cell lines the computation of the combination index was not possible. (0.69 MB PDF) [file pone.0008919.s002.pdf]

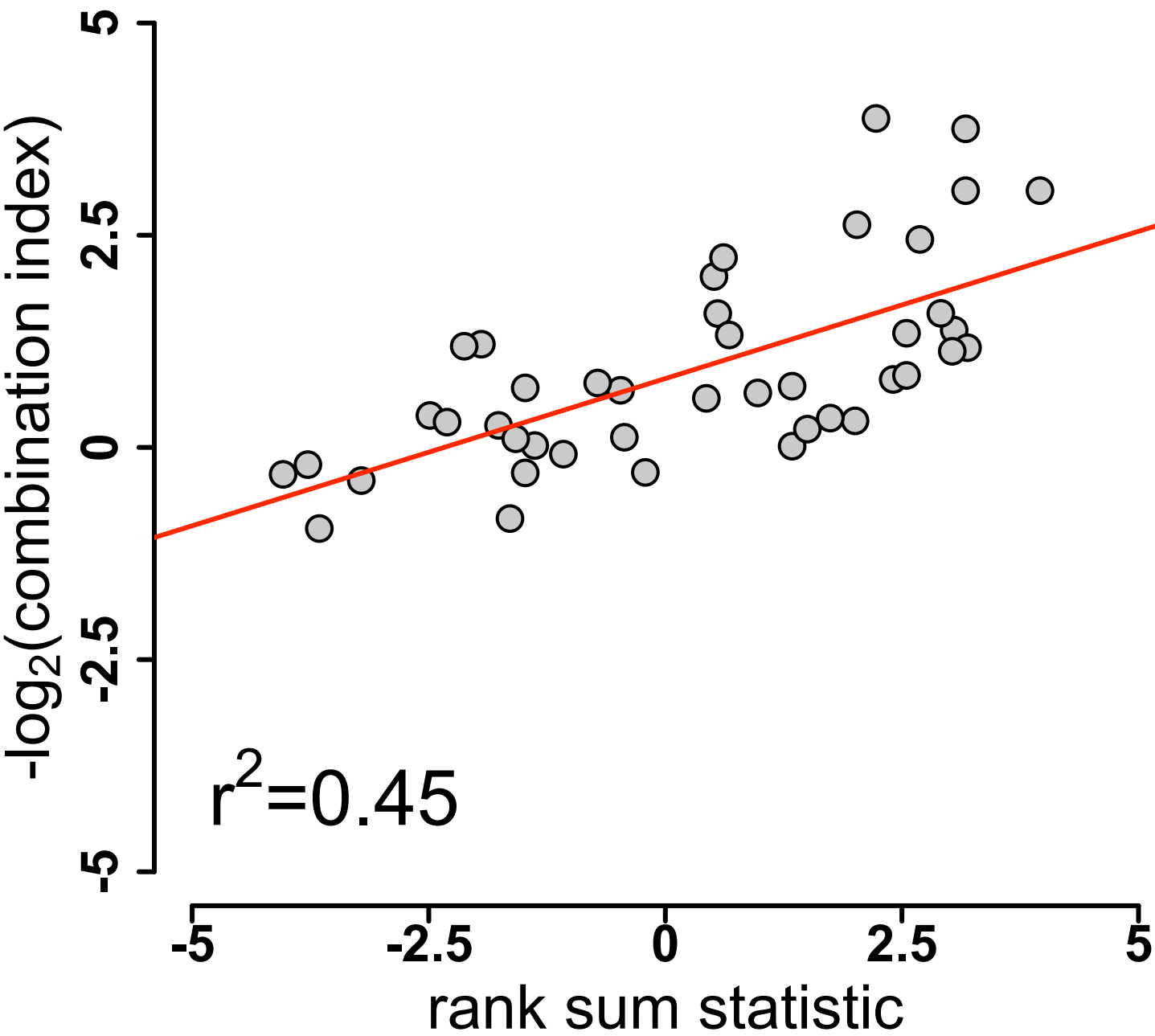

Supplement: Figure S2 — Correlation analysis between both methods. To adapt the scale of both measures, we performed a transformation of the combination index using the negative logarithm. The regression line is displayed by the straight red line. Moreover, we found a significant positive correlation (r2 = 0.45; p<10−6), which confirms that both methods follow the same trend. (0.12 MB PDF) [file pone.0008919.s003.pdf]
