# Supplementary material for: Analysis of Compound Synergy in High-Throughput Cellular Screens by Population-Based Lifetime Modeling
Source: PLoS One. 2010 Jan 27;5(1):e8919. doi: 10.1371/journal.pone.0008919 (PMC2811738; doi:10.1371/journal.pone.0008919)
Supplement: Table S1 — Genomic annotation of all 11 cell lines showing synergistic behavior. Significant copy number regions were identified using GISTIC. To assure comparability with mutation data, copy numbers were dichotomized with the following thresholds: 4 in case of amplifications and 1 for deletions. (0.03 MB PDF) [file pone.0008919.s004.pdf]

Supplementary Table S1

Cytoband	Lesion Type	Lesion	Colo699	EKVX	H1838	H1993	H2122	H2347	H2887	HCC2450	HCC366	HCC461	HCC78
1q21.3	amplification	RAB13, ARNT	0	0	0	1	0	0	0	0	1	0	0
1p22.1	amplification		0	0	0	0	0	0	0	0	0	0	0
3q26.2	amplification	PIK3CA	0	0	0	0	0	0	0	0	0	0	0
5p15.33	amplification	TERT	0	0	0	1	0	0	0	0	0	0	0
5p11	amplification		0	0	0	0	0	0	0	0	0	0	0
5p14.3	amplification	TRIO	0	0	0	0	0	0	0	0	0	0	0
6p21.2	amplification		0	0	0	0	0	0	0	0	1	0	0
7p11.2	amplification	EGFR	0	0	1	0	0	0	0	0	0	0	0
7p22.3	amplification		0	0	1	0	0	0	0	0	0	0	0
7q31.2	amplification		0	0	0	1	0	0	0	0	0	0	0
8q24.21	amplification	MYC	0	0	0	0	0	0	0	0	0	0	0
8p12	amplification		0	0	0	0	0	0	0	0	0	0	0
8q21.11	amplification		0	0	0	0	0	0	0	0	0	0	0
11q13.3	amplification	CCND1	0	1	0	0	0	0	0	0	0	0	0
11q22.3	amplification		0	1	0	1	0	0	0	0	0	0	0
11p13	amplification		0	0	0	0	0	0	0	0	0	0	0
12p11.23	amplification	ARNTL2, KRAS	1	0	0	0	0	0	1	0	0	1	0
12q14.1	amplification		0	0	0	0	0	0	0	0	0	0	0
12q12	amplification		0	0	0	1	0	0	0	0	0	0	0
14q13.3	amplification	MBIP, TITF1	0	0	0	0	0	0	0	0	0	0	0
17q21.1	amplification		0	0	0	0	0	0	0	0	0	0	0
19q13.2	amplification	MAPK4K1	0	0	0	0	0	0	1	0	0	0	0
19q13.31	amplification		0	0	0	0	0	0	0	0	0	0	0
19q13.11	amplification		0	0	0	0	0	0	0	0	0	0	0
20q11.22	amplification		0	0	0	0	0	0	0	0	0	0	0
20q11.21	amplification		0	0	0	0	0	0	0	0	0	0	0
20q13.33	amplification		0	0	0	0	0	0	0	0	0	0	0
20p12.1	amplification		0	0	0	0	0	0	0	0	0	0	0
22q11.21	amplification	CRKL, PIK4CA	1	0	0	0	0	0	0	0	0	0	0
2q22.1	deletion	LRP1B	0	0	0	0	1	0	0	0	0	0	0
3p14.2	deletion	FHIT	0	0	0	0	0	0	1	0	0	0	1
3p14.2	deletion	FHIT	0	1	0	0	0	0	0	0	0	0	0
3q13.31	deletion		0	0	0	0	0	0	0	0	0	0	0
3p26.1	deletion		0	0	0	1	0	0	0	0	0	0	0
3p14.2	deletion	FHIT	0	0	0	0	0	0	0	0	0	0	0
4q22.1	deletion		0	0	0	0	0	0	1	0	0	0	0
4q34.3	deletion		0	0	0	0	0	0	0	0	0	0	0
6p25.3	deletion		0	1	0	0	0	0	0	0	0	0	0
6q22.31	deletion		0	0	0	0	0	0	0	0	0	0	0
6q16.1	deletion	EPHA7	0	0	0	0	0	0	0	0	0	0	0
8p23.3	deletion	CSMD1	0	0	0	0	0	0	0	0	0	0	0
8p12	deletion		0	0	0	0	0	0	0	0	0	0	0
9q21.3	deletion	CDKN2A/CDKN2B	0	0	1	1	1	0	0	0	0	0	0
9p23	deletion	PTPRD	0	0	1	1	0	1	0	0	0	1	0
9p21.3	deletion		0	0	1	0	0	0	0	0	0	0	0
9p24.3	deletion		0	0	0	1	0	1	0	0	0	0	0
10p14	deletion		1	0	0	0	0	0	0	0	0	0	0
10q21.1	deletion		0	0	0	1	0	0	0	0	0	0	0
11q14.1	deletion		1	0	0	0	0	0	0	0	0	0	0
11p12	deletion		0	0	1	0	0	0	0	0	0	0	0
12q13.11	deletion		0	0	1	0	0	0	0	0	0	0	0
12p12.1	deletion		0	0	0	0	0	0	0	0	0	0	0
13q21.1	deletion		0	0	0	0	0	0	0	0	0	0	0
13q12.11	deletion		0	0	0	0	0	0	0	0	0	0	0
16q23.1	deletion	WWOX	0	0	0	0	0	0	1	0	0	0	0
17p12	deletion		0	0	0	0	0	1	0	0	0	0	0
18q22.3	deletion		0	0	0	0	0	0	0	0	0	1	0
18q12.1	deletion		0	0	1	0	0	0	1	0	0	0	0
18q12.3	deletion		0	0	1	0	0	0	0	0	0	0	0
20p12.2	deletion		0	0	0	0	0	0	0	0	0	0	0
20p11.22	deletion		0	0	0	0	0	0	0	0	0	0	0
21q11.2	deletion		0	0	0	0	0	0	0	0	1	0	0
21q21.1	deletion		0	0	1	0	0	0	0	0	1	0	0
12p12.1	mutation	KRAS	0	0	0	0	1	0	1	0	0	1	0
1p13.2	mutation	NRAS	0	0	0	0	0	1	0	0	0	0	0
7p11.2	mutation	EGFR	0	0	0	0	0	0	0	0	0	0	0
7q34	mutation	BRAF	0	0	0	0	0	0	0	0	0	0	0
3q23.3	mutation	PIK3CA	0	0	0	0	0	0	0	1	0	0	0
2p22	fusion gene	EML4ALK	0	0	0	0	0	0	0	0	0	0	0
10q23.3	mutation	PTEN	0	0	0	0	0	0	0	0	0	0	0
7p11.2	mutation	EGFR+T790M	0	0	0	0	0	0	0	0	0	0	0
