# Supplementary material for: Analysis of Compound Synergy in High-Throughput Cellular Screens by Population-Based Lifetime Modeling
Source: PLoS One. 2010 Jan 27;5(1):e8919. doi: 10.1371/journal.pone.0008919 (PMC2811738; doi:10.1371/journal.pone.0008919)
Supplement: Table S2 — Multi-lesion predictor of synergy tested with the KNN method, Fishers exact test and t-test are displayed; here, only p-values smaller than 5% are shown. The Youden-Index (i.e., sensitivity+specificity-1) of zero indicates that the result has no predictive power. (0.02 MB PDF) [file pone.0008919.s005.pdf]

Supplementary Table S2

Youden Index	Lesion type	Cytoband	Lesion	t-test	Fisher's exact test	Direction
**0**	deletion	21.q21.1		0.02	0.026	presence
	amplification	11q22.3			0.026	presence
	amplification	12p11.23	ARNTL2, KRAS		0.013	presence
	mutation	7p11.2	EGFR+T790M			abscence
